# Computational Methods for Image Analysis in Craniofacial Development and Disease

**DOI:** 10.1177/00220345241265048

**Published:** 2024-09-13

**Authors:** E. James, A.J. Caetano, P.T. Sharpe

**Affiliations:** 1 Centre for Oral Immunobiology and Regenerative Medicine, Barts and The London School of Medicine and Dentistry, Queen Mary University of London, London, UK; 2 Centre for Craniofacial and Regenerative Biology, Faculty of Dentistry, Oral and Craniofacial Sciences, King’s College London, London, UK

**Keywords:** spatial genomics, bioinformatics, multiomics, deep learning, advanced imaging, developmental biology

## Abstract

Observation is at the center of all biological sciences. Advances in imaging technologies are therefore essential to derive novel biological insights to better understand the complex workings of living systems. Recent high-throughput sequencing and imaging techniques are allowing researchers to simultaneously address complex molecular variations spatially and temporarily in tissues and organs. The availability of increasingly large dataset sizes has allowed for the evolution of robust deep learning models, designed to interrogate biomedical imaging data. These models are emerging as transformative tools in diagnostic medicine. Combined, these advances allow for dynamic, quantitative, and predictive observations of entire organisms and tissues. Here, we address 3 main tasks of bioimage analysis, image restoration, segmentation, and tracking and discuss new computational tools allowing for 3-dimensional spatial genomics maps. Finally, we demonstrate how these advances have been applied in studies of craniofacial development and oral disease pathogenesis.

## Introduction

**Figure 1.**
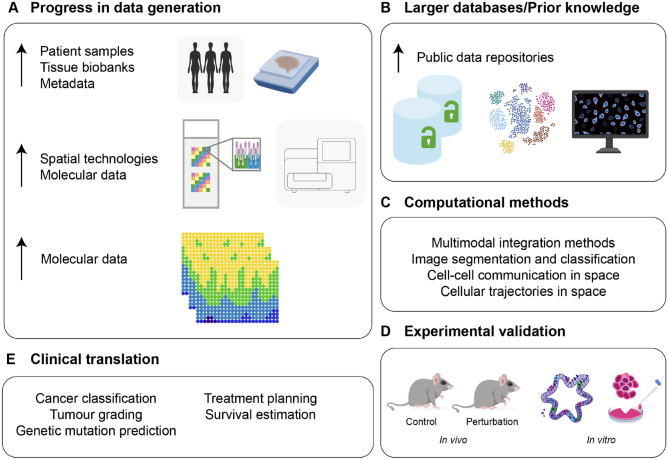
Technological progress in computational models and data access driving the development of novel imaging analysis tools. (**A**) Recent developments of spatial omics methods provide the ability to analyze archival clinical samples. (**B**) Increased access to open-access spatial data allows the development of novel computational methods. (**C**, **D**) High-throughput human spatial data inform meaningful functional validation using in vivo and in vitro model systems. (**E**) Analysis of high-throughput human spatial data has the potential to accelerate clinical translation.

Since its inception (Lovelace, 1842), computer programming has depended on biological learning. While biological systems have been an important source of inspiration in computing (Turing, 1947), perhaps more interesting is how applied numerical analysis can be used to study biological phenomena and develop novel experimental approaches. Recent years have seen a growing arrival of mathematicians, physicists, engineers, and computer scientists to the field of cell biology either to articulate sophisticated data or to model complex biological mechanisms. Collaborations like these are not new; developmental biologists often take inspiration from Thompson’s simple computations on growth and form (Thompson, 1917) or from Turing’s and Wolpert’s mathematical approaches to patterns and positional information for the development of new predictive models. However, until recently, there was a lack of high-quality data with which to feed and train models or architectures. With the increasing development of new experimental technologies and increased computing resources, this is no longer the case. Today, computational biology is a thriving field with many critical applications in biomedical research (Fig. 1A–D), including the field of oral and craniofacial biology, in which imaging and genomic analyses are already being used to understand craniofacial development, classify disease severity, and predict treatment outcomes (Fig. 1E).

In this review, we start on recent developments that have emerged in bioimage analysis using novel computational tools (image restoration, object detection/segmentation, and object quantification/tracking) as well as in spatial genomics analyses (multiple sample integration, cell communication, and cell trajectories in space). We further focus on 2 topics or biological applications that have recently benefited from advances in mechanistic thinking derived from these tools: first, organ development, and second, disease modeling and clinical practice. Understanding of these areas requires knowledge of cell interactions as well as cell localization under homeostatic conditions and in disease. Spatial information and advances in imaging tools are therefore essential to understand how a cell is influenced by its surrounding environment and how these interactions differ in homeostatic and disease states. This review aims to serve both as an introduction to clinicians and/or scientists on current available computational methodologies but also to help improve collaborations across distinct but ever complementing fields.

## Current Advances in Computational Microscopy and Sequencing Analysis

**Figure 2.**
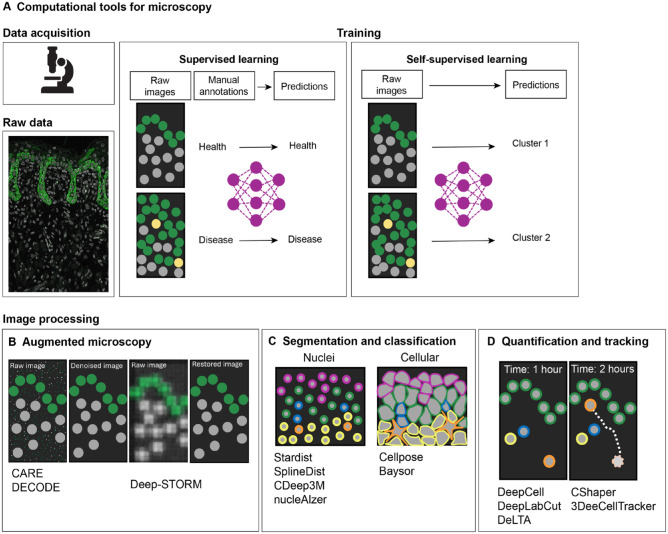
Computational tools for microscopy data analysis. Development of novel, problem-adapted, and robust machine learning approaches to extract quantitative biological information from microscopy images (**A**) have wide applications in imaging augmentation (**B**) (Weigert et al. 2018; Speiser et al. 2021), cell segmentation and classification (**C**) (Schmidt et al. 2018; Hollandi et al. 2020; Mandal and Uhlmann 2021; Petukhov et al. 2022), and cell quantification and tracking (**D**) (Van Valen et al. 2016; Cao et al. 2020; Lugagne et al. 2020; Wen et al. 2021).

Significant progress in the understanding of developmental and disease mechanisms is currently driven by the increased availability of complex image data. Since imaging data require quantification to be meaningful (Caicedo et al. 2017), there has been an increasing demand in the biosciences for new and problem-adapted processing tools, including deep learning (DL) methods to deal with the inherent complexity and volume of biological data (LeCun et al. 2015). Novel imaging processing tools allow automation of repetitive tasks and integration of large datasets to generate robust predictions. Development of some of these methods often requires training on large amounts of experimental data to construct annotated training datasets (supervised learning) for the subsequent deployment on new input data (unsupervised learning) to predict output labels (Fig. 2). Examples of unsupervised learning tasks include clustering and dimensionality reduction commonly used in single-cell genomics (Libbrecht and Noble 2015). Building trained algorithms that require minimal human input assumes a particular importance when analyzing highly variable datasets due to inherent biological differences (e.g., phenotypes) or technical parameters. Here, we describe recent computational tools for both microscopy data and sequencing-based imaging data.

### Microscopy Data Analysis

In bioimage analysis, DL methods are capable of extracting data features from images to perform specific tasks. Generally, these include image restoration, in which an input image is improved in resolution or denoised (Fig. 2B); image segmentation and classification, whereby an input image is divided into regions or objects of interest (Fig. 2C); and image quantification, in which objects can be classified and tracked (Fig. 2D). In recent years, DL has improved the robustness and accuracy of these tasks; for example, image restoration tools such as content-aware image restoration (Weigert et al. 2018) can extend the limits of what can be observed by microscopy. This DL-based image restoration algorithm was trained to resolve subdiffraction structures in low signal-to-noise ratio brightfield microscopy images using synthetically generated super-resolution data. Another image augmentation computational tool, DECODE (deep context dependent) is able to increase resolution in the context of single-molecule localization microscopy (Speiser et al. 2021). DL approaches have also greatly improved the speed and accuracy of object segmentation and detection in biological images; algorithms such as U-Net (Falk et al. 2019), Cellpose (Stringer et al. 2021), StarDist (Schmidt et al. 2018), SplineDist (Mandal and Uhlmann 2021), DeepCell (Van Valen et al. 2016), nucleAIzer (Hollandi et al. 2020), and Baysor (Petukhov et al. 2022) are capable of detecting and identifying nuclei shapes and cell membranes in challenging datasets. The automated segmentation of cell nuclei is routinely used in bioimage analysis; StarDist (Schmidt et al. 2018) significantly improved nuclei detection by being able to predict star-convex representations of object contours and can successfully separate overlapping nuclei in 2-dimensional (2D) images. Recently, with the increased availability of nuclei benchmarking datasets, more complex shapes are possible with SplineDist (Mandal and Uhlmann 2021). Cell membrane segmentation remains a challenge in the field; cells can take on varying morphologies with widely varying sizes and contour roughness. Generalist DL models trained on multiple datasets with a range of diverse morphologies, such as Cellpose, offer a significant advantage to other methods by being able to precisely segment cells from a wide range of image types in 2 and 3 dimensions (Stringer et al. 2021). DL methods have also been successfully applied to object quantification, used for counting, morphometry, or tracking. When manually performed, these tasks are time-consuming and have the potential for bias; therefore, DL methods are important and well poised to overcome these limitations. For example, DeepCell can automate tracking across entire populations of cells (Moen et al. 2019). This feature is particularly relevant in lineage tracing experiments in which tracking over long periods of time remains a challenge. To address this, DL-based lineage tracing methods, such as 3DeeCellTracker (Wen et al. 2021) and ELEPHANT (Sugawara et al. 2022) have been developed, which allow for the segmentation and tracking of cells in 3D. Training on large datasets allowed ELEPHANT to track cells over long periods of time. These tools are transforming experimental design and allowing quantitative and statistical analyses in an automated, unbiased, and high-throughput manner.

### Spatial Sequencing-Based Analysis

**Figure 3.**
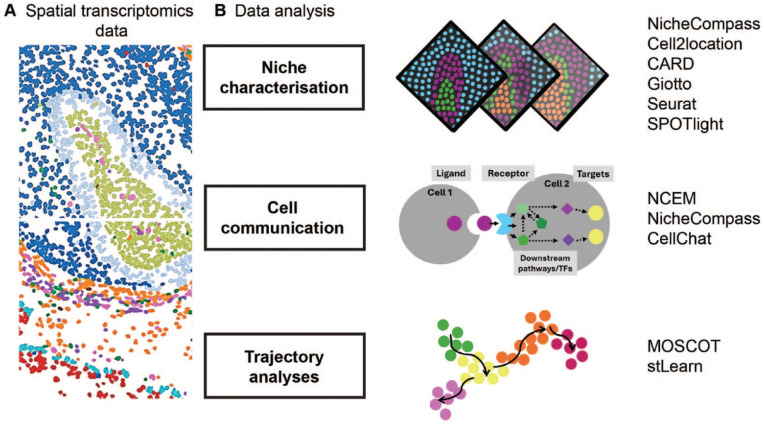
Computational tools for spatial transcriptomics analysis. (**A**) Representative image of imaging-based sequencing methods (CosMx, Nanostring). (**B**) Examples of computational tools for the analysis of spatial transcriptomics datasets. These allow improved integration and processing of multiple tissue samples, such as NicheCompass (Sebastian et al. 2024). It quantitatively describes cell niches based on cell-cell communication mechanisms, including metabolic and ligand-receptor interactions, while also providing a multimodal spatial profiling of gene expression and chromatin accessibility. Other recent analyses include modeling intercellular communication using spatial graphs of cells (Fischer et al. 2023) and trajectory inference, which incorporates spatial and lineage information (Dominik et al. 2023).

DL-based methods applied in microscopy analyses are also applied in spatial genomics data (Fig. 3). We previously discussed and summarized recent developments in spatial data generation and analysis for the study of oral mucosa homeostasis (Caetano and Sharpe 2024). Here, we focus on novel computational models applied to this data modality that have recently shifted our ability to study molecular dynamics of tissue organization and cellular differentiation in space. We discuss recent studies that address a common limitation of many imaging- and next-generation sequencing (NGS)–based techniques, which is their inherent 2D nature. To fully understand tissue function, 3-dimensional (3D) data are essential to explore the complete cellular microenvironment. Recent work has addressed this either through improved spatial integration algorithms or by reconstruction of the original 3D positions of cells in a tissue after tissue dissociation (Cotterell et al. 2024).

Several pipelines are now available to integrate multiple tissue samples to move on from 2D representations (Dries et al. 2021; Zhang et al. 2022; Guo et al. 2023; Zhou et al. 2023; Sebastian et al. 2024; Varrone et al. 2024; Yuan 2024). For example, NicheCompass (Niche Identification based on Cellular graph Embeddings of COMmunication Programs Aligned across Spatial Samples) is different from previous spatial omics pipelines as it is a generative graph DL method constructed on principles of cellular communication and incorporates multiple data modalities across distinct tissue samples and conditions (Sebastian et al. 2024). It is built on prior gene programs (GPs) to learn interpretable representations of cells or spots, but it can learn de novo GPs not contained in the training datasets. Given its capability on data integration, it will allow for a better insight into the cellular dynamics of tissue response in disease, particularly by allowing inference of cellular communication across integrated samples (Fig. 3B).

While data integration efforts are increasingly more robust, there are limitations of integrating multiple samples. First, there is the idea that tissue sections have the same geometric arrangement, and second, these are time-consuming tasks requiring extensive computational resources. Moreover, NGS-based techniques do not allow single-cell mapping between cells and spatial barcodes and require deconvolution tools to identify specific cell types at each location. To generate comprehensive 3D spatial maps, a novel technique enabling the preservation of the 3D location for each cell was developed. This method, Cell 3D Positioning by Optical encoding (C3PO), works with optical labels instead of positional barcodes (Cotterell et al. 2024). By generating gradients of fluorescence intensity throughout a tissue, it creates a coordinate system so that each individual cell carries an optical address of its original position within the tissue. Tagged cells can then be dissociated and pooled with their positions recovered by recording the 4 fluorescent channels before performing transcriptomics.

In addition to integration pipelines applied to spatial data, other common spatial analysis tasks such as inferring cell-cell communication and differentiation trajectories remain limited to the analysis of 2D tissue sections, except NicheCompass (Sebastian et al. 2024). Indeed, models to perform these tasks with spatial representation have been developed only recently (Fig. 3B). Such examples include node-centric expression models (NCEM) (Fischer et al. 2023), multiomics single-cell optimal transport (Moscot) (Dominik et al. 2023), and the newly updated CellChat (Jin et al. 2023). NCEM improve cell communication inference with spatial graphs of cells (Fischer et al. 2023). Other mathematical models of cell interactions in spatial data had failed to represent statistical dependencies of gene expression. Using this model, Fischer et al. discovered a bidirectional dependency of B cells and follicular dendritic cells in lymph nodes. Using organism-wide datasets, the authors could train cross-tissue models to predict cell type or niche composition at scale with high-impact applications from developmental biology to disease pathogenesis. Finally, to understand cellular trajectories in space, multiomics single-cell optimal transport (Moscot) was developed to allow for the reconstruction of time and space from optimal transport theory, an area of applied mathematics concerned with comparing and aligning probability distributions (Gabriel and Marco 2019; Villani 2009). Moscot can incorporate multimodal information and was used to recover murine differentiation trajectories during embryogenesis across time and space (Dominik et al. 2023). Combined with CellRank (Lange et al. 2022), this framework identified candidate regulators in heart formation.

Overall, these computational tools have led to improved detection and understanding of biological events, for example, in developmental biology and disease mechanisms as described next.

## Biological Applications of Computational-Based Imaging Models

### Organ Development and Tissue Homeostasis

Cell fate transition is fundamentally a spatiotemporal process; incorporating both space and time into models is essential to characterize interactions among neighboring cells. To explore this, a new approach was developed whereby a probabilistic encoder was linked to a temporal convolution network to predict the fate of each cell in an epithelium. Of note, the researchers worked toward creating a predictive model of cell fate that requires minimal human input and can predict cell fate prior to any recognizable morphological features of mitosis or apoptosis (the 2 outcomes of cell competition). Specifically, they trimmed the single-cell trajectories to remove these recognizable morphological features of cell fate. This model learnt that cell density is the primary predictor of cell fate, which corroborates previous work into cell competition (Soelistyo et al. 2022). This work highlights how predictive models can gain valuable insights from simplistic imaging data. Moreover, these results show promise for predictive models in oral research, which could prove useful in predicting cell fate in oral embryology and oral cancer biology.

The head is one of the most anatomically and functionally intricate structures. Advances in imaging technologies have always been central to understanding the complex and dynamic processes of craniofacial development. The ability to image and computationally reconstruct whole-embryo and -tissue development at the cellular level enables detailed analysis of such complex structures and morphodynamic events never visualized in vivo. McDole et al. (2018) developed an improved light-sheet microscope combined with a novel computational framework for reconstructing automated long-term cell tracking, detection of cell divisions, construction of high-resolution fate maps, and spatiotemporal maps of tissue morphodynamics across the entire mouse embryo. This work provided the first movie of mouse embryonic development at single-cell resolution from gastrulation to embryogenesis. It also pioneered the instruction of how to develop image analysis software that extracts quantitative information from vast amounts of data. More recently, combined whole-mount immunolabeling and light-sheet imaging were used to systematically analyze development of the human head in embryos (Blain et al. 2023). Embryos of gestational ages ranging from 5.5 to 13 weeks postconception were fixed and stained with various antibodies to investigate the development of the head skeleton, muscle, salivary and lacrimal glands, and arteries. After image acquisition from all planes of the sample, Blain et al. stitched the files to reconstruct 3D images. They next used a virtual reality software, syGlass, along with the Oculus Quest headset to segment anatomical structures. These segmented regions could be visualized as a mesh and, after more processing in Python, 3D printed. These methods, while providing remarkable advances in imaging techniques, remain limited by image resolution in live imaging settings or static 3D images. By using a mathematical approach, Dalmasso et al. took advantage of spherical harmonics (known since 1782, Laplace) to generate a computer-based approach to describe organ development by producing a quantitative 4-dimensional description of morphogenesis, including predictions of gradual changes in length and volumes of the tissue (Dalmasso et al. 2022). Overall, in common, these studies demonstrate how imaging combined with computational and mathematical approaches is transforming our ability to reconstruct the position and movements of individual cells to understand whole-organ development.

While knowledge of the precise timing and location of cellular and tissue-level processes is essential to study embryogenesis, understanding the spatiotemporal control of gene expression that drives cell fate choices is also indispensable. Spatial transcriptomics has begun to address these questions, and teams of researchers are building genomics maps of whole organs and tissues. Two main international collaborative networks dedicated to craniofacial research, the Human Cell Atlas (Caetano et al. 2022) and FaceBase (Samuels et al. 2020) aim to create reference maps throughout development and adulthood and provide open-access data resources.

Piña et al. (2023) recently investigated secondary palate development using spatially resolved RNA sequencing, identifying significant changes at the onset of osteogenesis. They defined regionalized patterns of gene expression of key osteogenic marker genes that are differentially expressed across developmental time (Piña et al. 2023). In another study, computational tools were applied to generate high-resolution temporal trajectories of secondary palate development (CellRank), which identified known driver genes involved in craniofacial development such as *Msx1*, *Runx2*, *Dlx1*, and *Dlx2* (Yan et al. 2024). Interestingly, the authors also applied an in silico perturbation model (Kamimoto et al. 2023) to test the impact of specific regulators of palate development; this identified *Shox2* and *Meox2* as driver genes in establishing anterior-posterior polarity. Importantly, this model was validated with experimental in vivo data, highlighting the robustness of the computational method. Spatial transcriptomics also allowed mapping changes in the molecular landscape across the developing cranium of mice (Tower et al. 2021); importantly, using a model in which sensory innervation is blocked, they were able to mechanistically determine how neuronal signaling regulates cranial bone patterning formation. Here, they further developed a method similar to pseudotime for trajectory analysis called SpatialTime (Tower et al. 2021).

While there are still limited studies applying imaging- or sequencing-based methods to understand craniofacial biology, these already provided essential mechanistic insights and highlight the usefulness and wide application of these data when combined with experimental evidence.

### Disease Modeling and Predictive Medicine

Computational tools based on DL methods are poised to be particularly useful in clinical practice, either for the better understanding of pathogenic mechanisms but also in clinical diagnosis and predictive medicine. Predictive medicine seeks to understand an individual’s disease susceptibility and predict treatment effectiveness. For predictive medicine to be useful in clinic, researchers first need to understand organ and tissue function at cellular or near-cellular resolution. Computational models trained with large datasets can now be used in histopathology for cell classifications, diagnosis, genetic mutation prediction, tumor grading, prognostication, or drug responses. Most progress made so far in these areas has been on histopathology and cancer biology; we now have genomic maps of tumors and practical applications of DL in medical image analysis (Bahadir et al. 2024). For example, Zaritsky et al. (2021) applied DL to analyze a dataset consisting of 1.7 million raw images, to classify patient-derived melanoma xenografts based on their metastasis efficiency. They collected label-free imaging data from patient-derived xenotransplantations as well as 2 untransformed melanocyte and 6 melanoma cell lines. Despite differences in metastatic potential, most cells displayed a similar rounded shape with dynamic surface features. Zaritsky’s team also trained an adversarial autoencoder (a deep convolutional neural network) to encode individual cell images into a latent vector and then decode them back into synthetic images. These generated images helped identify features associated with metastasis (Zaritsky et al. 2021). Similarly, Coudray et al. (2018) trained a deep convolutional neural network on whole-slide images to automatically classify adenocarcinomas, squamous cell carcinomas, and normal lung tissue.

Predictive models are being used not only to predict severity of disease but also to predict the risk of adverse effects from treatments. In head and neck cancer (HNC), a model was developed for predicting radiation-induced oral mucositis (OM) in HNC patients receiving radiotherapy (RT). Baseline computed tomography (CT) scan images were taken in 49 HNC patients prior to RT. CT images were delineated and segmented using the 3D slicer software, and PyRadiomics was used to extract radiomic features. Predictive models were then trained using a random forest algorithm with top-ranked features. This study suggests that it is possible to predict an individual’s risk of adverse effects from RT. Identification of individuals at risk of radiation-induced OM means early interventions can be implemented, lowering the incidence of OM (Agheli et al. 2024).

Visualizing the dynamic spatial architecture of the tumor microenvironment also provides essential insights into disease progression and treatment responses. Advances in not only spatial transcriptomics but also spatial proteomics and metabolomics and their combination have improved our understanding of pathogenic mechanisms. For example, multiplexed immunofluorescence performed on whole-slide serial sections from colorectal cancer provided a 3D atlas of cell-state transitions and cell interactions (Lin et al. 2023). This study concluded that molecular states and tissue morphologies are often graded, with phenotype changes ranging spatial scales. They also demonstrate that cellular communities studied in 2D at a local level are in fact organized into large, interconnected 3D structures. In one study by Arora et al. (2023), extensive characterization of 12 oral squamous cell carcinoma (OSCC) samples was performed using spatial transcriptomics integrated with HNC scRNA seq data. Deconvolution of ST data, haplotype-aware CNV inference, as well as pathologist annotations were used to characterize malignant versus nonmalignant spots. Unsupervised Louvain clustering was then performed on these malignant spots and identified spatially distinct regions, tumor core (TC), leading edge (LE), and transitionary cells. TC spots had higher expression of genes involved with keratinization, cell differentiation, and immune function, whereas LE spots indicated high expression of genes involved in epithelial-mesenchymal transition, angiogenesis, and the cell cycle. Interestingly, using machine-learning probability-based prediction models, the authors highlighted that this high LE score was associated with worse prognosis in other cancer types. In addition, the use of an in silico approach (Dynamo), which can accurately predict cell-fate transition, allowed for the prediction of therapeutic outcomes of various drugs targeting OSCC. These results highlight that ST data can be manipulated in silico, to identify potentially effective therapeutics as well as identify targets for therapeutic intervention (Arora et al. 2023).

## Conclusions

To summarize, we have reviewed novel computational approaches in imaging analysis for the generation of more informative representations of cells and tissues. We also highlight how these methods are contributing to rapid advances in developmental biology and clinical practice. While most of these methods have been applied outside the craniofacial complex, these technologies have wide applicability; for example, integration and analysis of multiple tissue samples with NicheCompass will allow a more comprehensive analysis of oral pathogenic mechanisms. Previously, we identified a fibroblast population localized in a highly immunogenic region of the oral mucosa (Caetano et al. 2023); however, we were able to integrate only 2 consecutive tissue sections, and ligand-receptor analysis in space could be performed only in separate samples. Thus, novel spatial integration tools will expand the robustness of these analyses and allow the identification of shared cell-communication patterns across patient samples for the identification of key dysregulated signaling pathways in disease. Moreover, the application of trajectory analysis with spatial embeddings will provide not only useful insights in oral developmental processes but also a better understanding of how these differentiation trajectories are affected in disease and contribute to irreversible changes in oral tissue architecture.

Computational tools discussed here also have the potential to improve clinical management of patients with various conditions. Current models used to generate more interpretable representations are still limited by data quality; thus, the more data are generated, the more we are able to integrate different types of measurements to develop better computational tools. DL methods trained on large-scale datasets are poised to change experimental approaches and affect clinical decision making; for example, spatial transcriptomics analysis of OSCC samples was able to characterize tumor regions to predict survival and treatment responses (Arora et al. 2023). Most oral cancer cases are diagnosed in advanced stages; therefore, these data have the potential to improve diagnostics and better inform treatment decision.

However, an important question remains: how to standardize these technologies for routine laboratory and clinical use. One solution could be to identify most informative data patterns (e.g., spatial features) through DL predictions and develop streamline pipelines that are accessible to biologists and clinicians that are cost-effective and time-efficient. Furthermore, technological advances move fast, leading to cost reduction and improved accessibility as demonstrated since the early days of whole genome studies.

A final consideration is the risk that among the increasing amount of data, we are missing well-established evidence from experimental work. While we agree that data collection and generation are essential for scientific discovery, the framing and biological conclusions are often neglected. In addition, ground truth data are often limited. As molecular biology becomes more predictive and quantitative and machine learning embedded in data mining or analysis, new ideas and biological theory will always be of most value. Closer collaboration between computational researchers, biologists, and clinicians will be essential to ensure that DL tools are clinically relevant and aligned with the needs of patients.

## Future Perspective

As spatial methods become more accessible, scalable, and robust, advances from basic to clinical research are likely to fast-track. We are already witnessing remarkable progress in areas such as oral cancer biology, contributing to the promise of improved diagnostics. However, training or developing DL models based on imaging and sequencing data is still computationally expensive and requires coding and math expertise, which hinders their routine adoption in most biomedical research laboratories. Several software tools now attempt to simplify their interface or provide pretrained data that do not need computational expertise (Hollandi et al. 2020; Stringer et al. 2021). Nevertheless, models perform best on data that are similar to training datasets; therefore, there is still a need to generate highly diverse training datasets to accommodate specific research models. Furthermore, most benchmark datasets are assembled in 2D; thus, construction of 3D equivalents would be essential to comprehensively study developmental and tissue biology. Finally, standardization of data acquisition, archival protocols, and metadata annotations need to be scientifically sound and informed for the creation of reliable predictive models.

## Author Contributions

E. James, contributed to conception, data acquisition and interpretation, drafted and critically revised the manuscript; A.J. Caetano, contributed to conception, design, data acquisition, drafted and critically revised the manuscript; P.T. Sharpe, contributed to conception, data acquisition, drafted and critically revised the manuscript. All authors have their final approval and agree to be accountable for all aspects of work.

## References

[bibr1-00220345241265048] Agheli R Siavashpour Z Reiazi R Azghandi S Cheraghi S Paydar R. 2024. Predicting severe radiation-induced oral mucositis in head and neck cancer patients using integrated baseline CT radiomic, dosimetry, and clinical features: a machine learning approach. Heliyon. 10(3):e24866. 10.1016/j.heliyon.2024.e24866 PMC10839875 38317933

[bibr2-00220345241265048] Arora R Cao C Kumar M Sinha S Chanda A McNeil R Samuel D Arora RK Matthews TW Chandarana S , et al. 2023. Spatial transcriptomics reveals distinct and conserved tumor core and edge architectures that predict survival and targeted therapy response. Nat Commun. 14(1):5029. 37596273 10.1038/s41467-023-40271-4 PMC10439131

[bibr3-00220345241265048] Bahadir CD Omar M Rosenthal J Marchionni L Liechty B Pisapia DJ Sabuncu MR . 2024. Artificial intelligence applications in histopathology. Nat Rev Electr Eng. 1(2):93–108.

[bibr4-00220345241265048] Blain R Couly G Shotar E Blevinal J Toupin M Favre A Abjaghou A Inoue M Hernandez-Garzon E Clarencon F , et al. 2023. A tridimensional atlas of the developing human head. Cell. 186(26):5910–5924.e17. 10.1016/j.cell.2023.11.013 PMC10783631 38070509

[bibr5-00220345241265048] Caetano AJ Human Cell Atlas O Craniofacial B Sequeira I Byrd KM . 2022. A roadmap for the human oral and craniofacial cell atlas. J Dent Res. 101(11):1274–1288. 36154725 10.1177/00220345221110768 PMC9516614

[bibr6-00220345241265048] Caetano AJ Redhead Y Karim F Dhami P Kannambath S Nuamah R Volponi AA Nibali L Booth V D’Agostino EM , et al. 2023. Spatially resolved transcriptomics reveals pro-inflammatory fibroblast involved in lymphocyte recruitment through CXCL8 and CXCL10. Elife. 12:e81525. 10.7554/eLife.81525 PMC9897724 36648332

[bibr7-00220345241265048] Caetano AJ Sharpe PT . 2024. Redefining mucosal inflammation with spatial genomics. J Dent Res. 103(2):129–137. 38166489 10.1177/00220345231216114 PMC10845836

[bibr8-00220345241265048] Caicedo JC Cooper S Heigwer F Warchal S Qiu P Molnar C Vasilevich AS Barry JD Bansal HS Kraus O , et al. 2017. Data-analysis strategies for image-based cell profiling. Nat Methods. 14(9):849–863. 28858338 10.1038/nmeth.4397 PMC6871000

[bibr9-00220345241265048] Cao J Guan G Ho VWS Wong M-K Chan L-Y Tang C Zhao Z Yan H. 2020. Establishment of a morphological atlas of the caenorhabditis elegans embryo using deep-learning-based 4D segmentation. Nat Commun. 11(1):6254. 33288755 10.1038/s41467-020-19863-x PMC7721714

[bibr10-00220345241265048] Cotterell J Swoger J Robert-Moreno A Cardona H Musy M Sharpe J. 2024. Cell 3D positioning by optical encoding (C3PO) and its application to spatial transcriptomics. bioRxiv. 10.1101/2024.08.11.606848

[bibr11-00220345241265048] Coudray N Ocampo PS Sakellaropoulos T Narula N Snuderl M Fenyö D Moreira AL Razavian N Tsirigos A. 2018. Classification and mutation prediction from non-small cell lung cancer histopathology images using deep learning. Nat Med. 24(10):1559–1567. 30224757 10.1038/s41591-018-0177-5 PMC9847512

[bibr12-00220345241265048] Dalmasso G Musy M Niksic M Robert-Moreno A Badía-Careaga C Sanz-Ezquerro JJ Sharpe J. 2022. 4D reconstruction of murine developmental trajectories using spherical harmonics. Dev Cell. 57(17):2140–2150.e5. 10.1016/j.devcel.2022.08.005 PMC9481268 36055247

[bibr13-00220345241265048] Dominik K Giovanni P Marius L Michal K Zoe P Manuel G Laetitia M-P Michael S Aimée B-P Marta T-M , et al. 2023. Mapping cells through time and space with moscot. bioRxiv [2023 May 11]. doi:https://10.1101/2023.05.11.540374

[bibr14-00220345241265048] Dries R Zhu Q Dong R Eng CL Li H Liu K Fu Y Zhao T Sarkar A Bao F , et al. 2021. Giotto: a toolbox for integrative analysis and visualization of spatial expression data. Genome Biol. 22(1):78. 33685491 10.1186/s13059-021-02286-2 PMC7938609

[bibr15-00220345241265048] Falk T Mai D Bensch R Çiçek Ö Abdulkadir A Marrakchi Y Böhm A Deubner J Jäckel Z Seiwald K , et al. 2019. U-net: deep learning for cell counting, detection, and morphometry. Nat Methods. 16(1):67–70. 30559429 10.1038/s41592-018-0261-2

[bibr16-00220345241265048] Fischer DS Schaar AC Theis FJ . 2023. Modeling intercellular communication in tissues using spatial graphs of cells. Nat Biotechnol. 41(3):332–336. 36302986 10.1038/s41587-022-01467-z PMC10017508

[bibr17-00220345241265048] Gabriel P Marco C. 2019. Computational optimal transport: with applications to data science. now. Foundations and Trends® in Machine Learning. 11(5–6):355–607. doi:10.1561/2200000073

[bibr18-00220345241265048] Guo T Yuan Z Pan Y Wang J Chen F Zhang MQ Li X. 2023. Spiral: integrating and aligning spatially resolved transcriptomics data across different experiments, conditions, and technologies. Genome Biol. 24(1):241. 37864231 10.1186/s13059-023-03078-6 PMC10590036

[bibr19-00220345241265048] Hollandi R Szkalisity A Toth T Tasnadi E Molnar C Mathe B Grexa I Molnar J Balind A Gorbe M , et al. 2020. Nucleaizer: a parameter-free deep learning framework for nucleus segmentation using image style transfer. Cell Systems. 10(5):453–458.e6. 10.1016/j.cels.2020.04.003 PMC8247631 34222682

[bibr20-00220345241265048] Jin S Plikus MV Nie Q. 2023. CellChat for systematic analysis of cell-cell communication from single-cell and spatially resolved transcriptomics. bioRxiv [2023 Nov 5]. doi:10.1101/2023.11.05.565674 39289562

[bibr21-00220345241265048] Kamimoto K Stringa B Hoffmann CM Jindal K Solnica-Krezel L Morris SA . 2023. Dissecting cell identity via network inference and in silico gene perturbation. Nature. 614(7949):742–751. 36755098 10.1038/s41586-022-05688-9 PMC9946838

[bibr22-00220345241265048] Lange M Bergen V Klein M Setty M Reuter B Bakhti M Lickert H Ansari M Schniering J Schiller HB , et al. 2022. Cellrank for directed single-cell fate mapping. Nat Methods. 19(2):159–170. 35027767 10.1038/s41592-021-01346-6 PMC8828480

[bibr23-00220345241265048] LeCun Y Bengio Y Hinton G. 2015. Deep learning. Nature. 521(7553):436–444. 26017442 10.1038/nature14539

[bibr24-00220345241265048] Libbrecht MW Noble WS . 2015. Machine learning applications in genetics and genomics. Nat Rev Genet. 16(6):321–332. 25948244 10.1038/nrg3920 PMC5204302

[bibr25-00220345241265048] Lin JR Wang S Coy S Chen YA Yapp C Tyler M Nariya MK Heiser CN Lau KS Santagata S , et al. 2023. Multiplexed 3D atlas of state transitions and immune interaction in colorectal cancer. Cell. 186(2):363–381.e19. 10.1016/j.cell.2022.12.028 PMC10019067 36669472

[bibr26-00220345241265048] Lugagne JB Lin H Dunlop MJ . 2020. Delta: automated cell segmentation, tracking, and lineage reconstruction using deep learning. PLoS Comput Biol. 16(4):e1007673. 10.1371/journal.pcbi.1007673 PMC7153852 32282792

[bibr27-00220345241265048] Lovelace A . 1842. Sketch of the analytical engine created by Charles Babbage. http://www.fourmilab.ch/babbage/sketch.html

[bibr28-00220345241265048] Mandal S Uhlmann V. 2021. Splinedist: automated cell segmentation with spline curves. Presented at the 2021 IEEE 18th International Symposium on Biomedical Imaging (ISBI); 2021; Nice, France. p. 1082–1086. doi:10.1109/ISBI48211.2021.9433928

[bibr29-00220345241265048] McDole K Guignard L Amat F Berger A Malandain G Royer LA Turaga SC Branson K Keller PJ . 2018. In toto imaging and reconstruction of post-implantation mouse development at the single-cell level. Cell. 175(3):859–876.e33. 10.1016/j.cell.2018.09.031 30318151

[bibr30-00220345241265048] Moen E Bannon D Kudo T Graf W Covert M Van Valen D. 2019. Deep learning for cellular image analysis. Nat Methods. 16(12):1233–1246. 31133758 10.1038/s41592-019-0403-1 PMC8759575

[bibr31-00220345241265048] Petukhov V Xu RJ Soldatov RA Cadinu P Khodosevich K Moffitt JR Kharchenko PV . 2022. Cell segmentation in imaging-based spatial transcriptomics. Nat Biotechnol. 40(3):345–354. 34650268 10.1038/s41587-021-01044-w

[bibr32-00220345241265048] Piña JO Raju R Roth DM Winchester EW Chattaraj P Kidwai F Faucz FR Iben J Mitra A Campbell K , et al. 2023. Multimodal spatiotemporal transcriptomic resolution of embryonic palate osteogenesis. Nat Commun. 14(1):5687. 37709732 10.1038/s41467-023-41349-9 PMC10502152

[bibr33-00220345241265048] Samuels BD Aho R Brinkley JF Bugacov A Feingold E Fisher S Gonzalez-Reiche AS Hacia JG Hallgrimsson B Hansen K , et al. 2020. Facebase 3: analytical tools and fair resources for craniofacial and dental research. Development. 147(18):dev191213. 10.1242/dev.191213 PMC7522026 32958507

[bibr34-00220345241265048] Schmidt U Weigert M Broaddus C Myers G. (2018). Cell detection with star-convex polygons. In: Frangi A Schnabel J Davatzikos C , editors. Medical Image Computing and Computer Assisted Intervention—MICCAI 2018. MICCAI 2018. Lecture Notes in Computer Science, Vol 11071. Cham (UK): Springer. doi:10.1007/978-3-030-00934-2_30

[bibr35-00220345241265048] Sebastian B Irene B-P Adib Miraki F Adam B Eneritz A Fani M Anna M Rong F Gonçalo C-B Omer Ali B , et al. 2024. Large-scale characterization of cell niches in spatial atlases using bio-inspired graph learning. bioRxiv [2024 Feb 23]. doi:10.1101/2024.02.21.581428

[bibr36-00220345241265048] Soelistyo CJ Vallardi G Charras G Lowe AR . 2022. Learning biophysical determinants of cell fate with deep neural networks. Nat Mach Intell. 4:636–644. doi:10.1038/s42256-022-00503-6

[bibr37-00220345241265048] Speiser A Müller L-R Hoess P Matti U Obara CJ Legant WR Kreshuk A Macke JH Ries J Turaga SC . 2021. Deep learning enables fast and dense single-molecule localization with high accuracy. Nat Methods. 18(9):1082–1090. 34480155 10.1038/s41592-021-01236-x PMC7611669

[bibr38-00220345241265048] Stringer C Wang T Michaelos M Pachitariu M. 2021. Cellpose: a generalist algorithm for cellular segmentation. Nat Methods. 18(1):100–106. 33318659 10.1038/s41592-020-01018-x

[bibr39-00220345241265048] Sugawara K Çevrim Ç Averof M. 2022. Tracking cell lineages in 3D by incremental deep learning. eLife. 11:e69380. 10.7554/eLife.69380 PMC8741210 34989675

[bibr40-00220345241265048] Thompson DW . 1917. On growth and form. 1st ed. Cambridge (UK): Cambridge University Press.

[bibr41-00220345241265048] Tower RJ Li Z Cheng YH Wang XW Rajbhandari L Zhang Q Negri S Uytingco CR Venkatesan A Zhou FQ , et al. 2021. Spatial transcriptomics reveals a role for sensory nerves in preserving cranial suture patency through modulation of BMP/TGF-β signaling. Proc Natl Acad Sci U S A. 118(42):e2103087118. 10.1073/pnas.2103087118 PMC8545472 34663698

[bibr42-00220345241265048] Turing AM . 1952. The chemical basis of morphogenesis. Philos Trans R Soc Lond B Biol Sci. 237(641):37–72. 10.1098/rstb.2014.0218 PMC4360114 25750229

[bibr43-00220345241265048] Van Valen DA Kudo T Lane KM Macklin DN Quach NT DeFelice MM Maayan I Tanouchi Y Ashley EA Covert MW . 2016. Deep learning automates the quantitative analysis of individual cells in live-cell imaging experiments. PLoS Comput Biol. 12(11):e1005177. 10.1371/journal.pcbi.1005177 PMC5096676 27814364

[bibr44-00220345241265048] Varrone M Tavernari D Santamaria-Martínez A Walsh LA Ciriello G. 2024. Cellcharter reveals spatial cell niches associated with tissue remodeling and cell plasticity. Nat Genet. 56(1):74–84. 38066188 10.1038/s41588-023-01588-4

[bibr45-00220345241265048] Villani C . 2009. Stability of optimal transport. In: Villani C , editor. Optimal transport: old and new. Berlin: Springer Berlin Heidelberg. p. 773–793.

[bibr46-00220345241265048] Weigert M Schmidt U Boothe T Müller A Dibrov A Jain A Wilhelm B Schmidt D Broaddus C Culley S , et al. 2018. Content-aware image restoration: pushing the limits of fluorescence microscopy. Nat Methods. 15(12):1090–1097. 30478326 10.1038/s41592-018-0216-7

[bibr47-00220345241265048] Wen C Miura T Voleti V Yamaguchi K Tsutsumi M Yamamoto K Otomo K Fujie Y Teramoto T Ishihara T , et al. 2021. 3DeeCellTracker, a deep learning-based pipeline for segmenting and tracking cells in 3D time lapse images. eLife. 10:e59187. 10.7554/eLife.59187 PMC8009680 33781383

[bibr48-00220345241265048] Yan F Suzuki A Iwaya C Pei G Chen X Yoshioka H Yu M Simon LM Iwata J Zhao Z. 2024. Single-cell multiomics decodes regulatory programs for mouse secondary palate development. Nat Commun. 15(1):821. 38280850 10.1038/s41467-024-45199-x PMC10821874

[bibr49-00220345241265048] Yuan Z . 2024. Mender: fast and scalable tissue structure identification in spatial omics data. Nat Commun. 15(1):207. 38182575 10.1038/s41467-023-44367-9 PMC10770058

[bibr50-00220345241265048] Zaritsky A Jamieson AR Welf ES Nevarez A Cillay J Eskiocak U Cantarel BL Danuser G. 2021. Interpretable deep learning uncovers cellular properties in label-free live cell images that are predictive of highly metastatic melanoma. Cell Systems. 12(7):733–747.e6. 10.1016/j.cels.2021.05.003 PMC8353662 34077708

[bibr51-00220345241265048] Zhang X Wang X Shivashankar GV Uhler C. 2022. Graph-based autoencoder integrates spatial transcriptomics with chromatin images and identifies joint biomarkers for Alzheimer’s disease. Nat Commun. 13(1):7480. 36463283 10.1038/s41467-022-35233-1 PMC9719477

[bibr52-00220345241265048] Zhou X Dong K Zhang S. 2023. Integrating spatial transcriptomics data across different conditions, technologies and developmental stages. Nat Comput Sci. 3(10):894–906. 38177758 10.1038/s43588-023-00528-w

